# Real world propensity score matched analysis evaluating the influence of en-bloc vs. non en-bloc techniques, energy and instrumentation on enucleation outcomes for large and very large prostates

**DOI:** 10.1007/s00345-024-04959-6

**Published:** 2024-05-06

**Authors:** Patrick Juliebø-Jones, Vineet Gauhar, Daniele Castellani, Khi Yung Fong, Mario Sofer, Marek Zawadzki, Nariman Gadzhiev, Giacomo Maria Pirola, Abhay D. Mahajan, Pankaj Nandkishore Maheshwari, Vigen Malkhasyan, Sarvajit Biligere, Mehmet İlker Gökce, Luigo Cormio, Dmitry Enikeev, Fernando Gómez Sancha, Thomas R. W. Herrmann, Bhaskar K. Somani

**Affiliations:** 1 Department of Clinical Medicine, University of Bergen, Bergen, Norway; 2 Department of Urology, Haukeland University Hospital, Bergen, Norway; 3 Department of Urology, University Hospital Southampton, Southampton, UK; 4 Department of Urology, Ng Teng Fong Hospital, Singapore, Singapore; 5 Urology Division, Azienda Ospedaliero-Universitaria Ospedali Riuniti Di Ancona, Università Politecnica Delle Marche, Ancona, Italy; 6 Yong Loo Lin School of Medicine, National University of Singapore, Singapore, Singapore; 7 Department of Urology, Sackler Faculty of Medicine, Tel-Aviv Sourasky Medical Center, Tel-Aviv University, Tel-Aviv, Israel; 8 Urology Unit, St. Anna Hospital, Piaseczno, Poland; 9 Department of Urology, Saint-Petersburg State University Hospital, Saint-Petersburg, Russia; 10 Urology Unit, IRCCS Multimedica, Multimedica Group, San Giuseppe Hospital, Milan, Italy; 11 Sai Urology Hospital and Mahatma Gandhi Mission’s Medical College and Hospital, Aurangabad, India; 12 Urology Unit, Fortis Hospital Mulund, Mumbai, India; 13 Urology Unit, A.I. Evdokimov Moscow State University of Medicine and Dentistry, Moscow, Russia; 14 Department of Urology, Ankara University School of Medicine, Ankara, Turkey; 15 Andrology and Urology Unit, Bonomo Teaching Hospital, Andria, Italy; 16 Department of Urology, Ospedali Riuniti di Foggia, University of Foggia, Foggia, Italy; 17 Department of Urology, Medical University of Vienna, Vienna, Austria; 18 Urology Department, Rabin Medical Center, Petah Tikva, Israel; 19 Institute for Urology and Reproductive Health, Sechenov University, Moscow, Russia; 20 Department of Urology and Robotic Surgery, ICUA-Clínica CEMTRO, Madrid, Spain; 21 Department of Urology, Kantonspital Frauenfeld, Spital Thurgau AG, Frauenfeld, Switzerland; 22 Department of Clinical Medicine, Hannover Medical School, Hannover, Germany

**Keywords:** Prostatic hyperplasia, Endoscopic enucleation of the prostate, Laser therapy, Complications, Urinary incontinence

## Abstract

**Purpose:**

The primary aim of the study was to evaluate if en-bloc vs. non en-bloc made a difference to intra-, peri- and post-operative surgical outcomes of anatomical endoscopic enucleation (AEEP) in large (> 80 cc) and very large prostates (> 200 cc). The secondary aim was to determine the influence of energy and instruments used.

**Methods:**

Data of patients with > 80 cc prostate who underwent surgery between 2019 and 2022 were obtained from 16 surgeons across 13 centres in 9 countries. Propensity score matching (PSM) was used to reduce confounding. Logistic regression was performed to evaluate factors associated with postoperative urinary incontinence (UI).

**Results:**

2512 patients were included with 991 patients undergoing en-bloc and 1521 patients undergoing non-en-bloc. PSM resulted in 481 patients in both groups. Total operation time was longer in the en-bloc group (*p* < 0.001), enucleation time was longer in the non en-bloc group (*p* < 0.001) but morcellation times were similar (*p* = 0.054). Overall, 30 day complication rate was higher in the non en-bloc group (16.4% vs. 11.4%; *p* = 0.032). Rate of late complications (> 30 days) was similar (2.3% vs. 2.5%; *p* > 0.99). There were no differences in rates of UI between the two groups. Multivariate analysis revealed that age, Qmax, pre-operative, post-void residual urine (PVRU) and total operative time were predictors of UI.

**Conclusions:**

In experienced hands, AEEP in large prostates by the en-bloc technique yields a lower rate of complication and a slightly shorter operative time compared to the non en-bloc approach. However, it does not have an effect on rates of post-operative UI.

## Introduction

Surgical treatment benign prostatic obstruction (BPO) can be categorised into resection, enucleation, vaporisation, alternative ablative techniques and non-ablative techniques [1]. Large (> 80 cc) prostate size often presents a heightened surgical challenge and the therapeutic options at the surgeon’s disposal have historically been more limited [2]. However, enucleation methods represent an alternative that can yield improved efficacy and safety compared to the traditional standard of open simple prostatectomy (OSP). Since the early description from Hiraoka in 1983, enucleation methods have undergone many developments [3, 4]. There are now a multitude of variations available that can be grouped under the umbrella term of anatomical endoscopic enucleation (AEEP) [5]. Practice patterns vary and there still remain unanswered questions regarding the role of different techniques (e.g., classic three lobe vs. en-bloc) on the outcomes associated with AEEP. Proposed advantages of the en-bloc method include superior visibility and easier identification of the surgical capsule, which can facilitate dissection in the correct plane [6]. Other areas of debate include optimal energy sources (e.g., bipolar vs. laser based) and instrument choices (e.g. scope size) [7, 8]. Furthermore, and as highlighted by the European guidelines, there remains a lack of high level evidence on surgical outcomes for prostates > 80 cc [1]. Further studies evaluating this subject area are, therefore, needed.

The primary aim of the study was to evaluate if the choice of technique, namely en-bloc vs. non en-bloc (2 or 3 lobe) made a difference to intra-, peri- and post-operative surgical outcomes of anatomical endoscopic enucleation (AEEP) in large (> 80 cc) and very large prostates (> 200 cc). The secondary aim was to determine the influence of energy and instruments used.

## Methods

### Registry design and enrolment protocol

The “Prostate Endoscopic Enucleation” (PEEL) registry is a retrospective multicentre anonymised database of patients with clinically diagnosed BPO with large prostates (> 80 cc in volume) undergoing enucleation. Institutional review board approval was obtained by Asian Institute of Nephrology and Urology, Hyderabad (AINU #11/2022), which was the main centre.

### Study population

Data of patients who underwent surgery between 2019 and 2022 for BPO were obtained from 16 surgeons across 13 centres in 9 countries. Only experienced surgeons having performed at least 200 cases of enucleation were invited to contribute. Exclusion criteria includes previous surgery of the prostate and/or urethra, prostate cancer and pelvic radiotherapy. Local protocols of the respective instituitions determined antibiotic prophylaxis. Prostate volume was determined by surgeon preferences and included US, CT and or MRI. En bloc technique referred to the original technique of Saitta et al. and included the early apical release. (EAR) [9]. Non-en bloc referred to the procedure being done by either a 2 or 3 lobe technique.

### Patient follow-up and secondary treatment

Follow-up time points were 3, 6, 12, 24 months. For this study, the definition used for incontinence was any urinary leakage reported by the patients.

### Outcome measures of interest

Primary: postoperative incontinence associated with en bloc and non-en bloc enucleation.

Secondary: early (≤ 30 days) and late (> 30 days) adverse events.

### Statistical analyses

Statistical analyses were carried out with R Statistical language, version 4.3.0 (R Foundation for Statistical Computing, Vienna, Austria). *p* < 0.05 indicated statistical significance with the Shapiro–Wilk test employed to assess for normality. Fisher exact test or χ^2^ test was used to compare for categorical parameters. Mann–Whitney *U* test was applied for continuous variables.

Propensity score matching (PSM) was used to reduce confounding. The following variables were included for matching: age, prostate volume, preoperative IPSS, preoperative Qmax, and preoperative PVR. To establish favourable matching, an absolute standardised mean difference (ASMD) threshold of < 0.1 was employed. Univariate analysis (UVA) was performed in order to evaluate factors associated with postoperative urinary incontinence and a multivariable model was built thereafter.

## Results

In total, 2512 patients were included. The sample comprised of 991 patients and 1521 patients undergoing non-en bloc and en bloc, respectively (Table 1). In this unmatched cohort, there were significant differences in terms of baseline characteristics such as age, prostate volume, IPSS and baseline cystometry findings. In contrast, PSM and resulted in 482 patients in both groups and revealed well-matched samples and follow-up data were available for all patients.

**Table 1:** Baseline characteristics

	Unmatched cohort			PSM cohort		
	Non-en-bloc (*N* = 991)	En-bloc (*N* = 1521)	ASMD	Non-en-bloc (*N* = 482)	En-bloc (*N* = 482)	ASMD
Age, median [IQR]	68 [63, 73.5]	70 [64, 75]	0.202	68 [63, 73]	68 [63, 74]	0.022
Prostate volume (ml), *n* (%)						
80–100	121 (12.2)	365 (24.0)	0.315	23 (4.8)	17 (3.5)	0.063
101–200	777 (78.4)	1053 (69.2)		413 (85.7)	419 (86.9)	
> 200	93 (9.4)	103 (6.8)		46 (9.5)	46 (9.5)	
Preoperative indwelling catheter, *n* (%)	162 (16.3)	336 (22.1)	0.146	48 (10.0)	29 (6.0)	0.146
Preoperative IPSS, median [IQR]	23 [21, 26]	26 [22, 29]	0.409	25 [22, 27]	25 [22, 29]	0.096
Preoperative QOL, median [IQR]	4.0 [4.0, 5.0]	5.0 [4.0, 5.0]	0.672	5.0 [4.0, 5.0]	5.0 [4.0, 5.0]	0.367
Preoperative Qmax, median [IQR]	8.7 [7.0, 11]	7.8 [6.0, 9.0]	0.447	8.0 [6.6, 9.8]	8.0 [6.4, 9.3]	0.027
Preoperative PVRU, median [IQR]	70 [50, 90]	69 [57, 110]	0.222	70 [54, 90]	68 [50, 90]	0.008

*IPSS *International prostate symptom score, *QOL *quality of life, *Qmax *maximum flow rate, *PVRU *post void residual volume

Regarding intra-operative characteristics, analysis of the PSM cohort revealed that a significantly higher number of patients had an operation with a 26Fr scope in the en-bloc group but this was the preferred scope for both cohorts. The en-bloc group had a greater proportion undergoing EAR (96.7% vs. 42.1%; *p* < 0.001) (Table 2). Interestingly, even in the non en-bloc group, 42.1% patients underwent EAR. The total operation time was only slightly longer in the en-bloc group but this did reach statistical significance (82 min, IQR 42–106 vs. 80 min, IQR 57–120; *p* < 0.001). Enucleation time was longer in the non en-bloc group (10 min, IQR 35–100 vs. 60 min, IQR 29–79; *p* < 0.001) but morcellation times were similar (15 min, IQR 10–25 vs. 18 min, IQR 12.5–25; *p* = 0.054). There was marked variation noted in the energy devices employed between the two groups in the PSM cohort. For non en-bloc, the two commonest devices used were Thulium fiber laser (TFL) (45.6%) and High-power Holmium laser (32.6%), whereas for en-bloc, it was High-power Holmium laser (47.9%) followed by bipolar enucleation (34.2%). Regarding post-operative outcomes, there were no differences in the duration of the indwelling catheter (2 days, IQR 1–2 vs. 2 days, IQR 1–2; *p* = 0.062) (Table 3). The overall 30 day complication rate in the PSM cohort was higher in the non en-bloc group (16.4% vs. 11.4%; *p* = 0.032). The commonest complication in the latter group was urinary tract infection (UTI), occurring in 6% compared to 0.6% in the en-bloc group (*p* < 0.001). However, the proportion requiring prolonged irrigation > 24 h for haematuria (Clavien II) was higher in the en-bloc group (5.8% vs. 2.7%; *p*: 0.025). There were two Clavien IV complications recorded in the non en-bloc group but none in the en-bloc group. The rate of late complications (> 30 days) was similar between the two groups (2.3% vs. 2.5%; *p* > 0.99). Only 16% of the en-bloc cohort experienced post-operative UI vs 17.8% of non en-bloc cohort but there was no significant difference between the groups (*p* = 0.492) However, of the patients with SUI (SUI), it was notably higher in the non en-bloc group (11.6 vs. 8.1; *p* = 0.084).

**Table 2:** Intraoperative characteristics

	Unmatched cohort			PSM cohort		
	Non-en-bloc (*N* = 991)	En-bloc (*N* = 1521)	*p*	Non-en-bloc (*N* = 482)	En-bloc (*N* = 482)	*p*
Scope size (Fr), *n* (%)						
22	0	53 (3.5)	< 0.001	0	0	< 0.001
24	142 (14.3)	0		83 (17.2)	0	
26	837 (84.5)	1435 (94.3)		392 (81.3)	461 (95.6)	
27	12 (1.2)	33 (2.2)		7 (1.5)	21 (4.4)	
Device energy, *n* (%)						
Low-power holmium laser	55 (5.5)	25 (1.6)	< 0.001	24 (5.0)	15 (3.1)	< 0.001
High-power holmium laser	301 (30.4)	926 (60.9)		157 (32.6)	231 (47.9)	
Holmium laser with MOSES	37 (3.7)	78 (5.1)		13 (2.7)	27 (5.6)	
Thulium fiber	424 (42.8)	46 (3.0)		220 (45.6)	30 (6.2)	
Thulium-YAG	46 (4.6)	27 (1.8)		5 (1.0)	14 (2.9)	
Bipolar enucleation	118 (11.9)	302 (19.9)		53 (11.0)	165 (34.2)	
Monopolar enucleation	10 (1.0)	0		10 (2.1)	0	
Virtual basket	0	117 (7.7)		0	0	
Early apical release, *n* (%)				203 (42.1)	466 (96.7)	< 0.001
Total operation time, median [IQR])	353 (35.6)	1492 (98.1)	< 0.001	80 [57, 120]	82 [42, 106]	< 0.001
Enucleation time, median [IQR])	85 [60, 120]	80 [53, 109]	< 0.001	70 [35, 100]	60 [29, 79]	< 0.001
Morcellation time, median [IQR])	75 [45, 100]	51 [30, 77]	< 0.001	15 [10, 25]	18 [12.5, 25]	0.054
Morcellator, *n* (%)						
Cyberblade	64 (6.8)	6 (0.4)	< 0.001	46 (10.1)	4 (0.8)	< 0.001
Hawk	1 (0.1)	0		0	0	
Jena	101 (10.8)	322 (21.2)		81 (17.8)	216 (44.8)	
Lumenis	37 (3.9)	11 (0.7)		7 (1.5)	9 (1.9)	
Piranha	595 (63.4)	1078 (70.9)		266 (58.6)	247 (51.2)	
Storz	141 (15.0)	104 (6.8)		54 (11.9)	6 (1.2)	
Spinal anaesthesia, *n* (%)	477 (48.1)	535 (35.2)	< 0.001	200 (41.5)	170 (35.3)	0.055

**Table 3:** Outcomes

	Unmatched cohort			PSM cohort		
	Non-en-bloc (*N* = 991)	En-bloc (*N* = 1521)	*p*	Non-en-bloc (*N* = 482)	En-bloc (*N* = 482)	*p*
Postoperative IDC duration (days), median [IQR]	2.0 [1.0, 2.0]	1.6 [1.0, 2.0]	< 0.001	2.0 [1.0, 2.0]	2.0 [1.0, 2.0]	0.062
30-day complications, *n* (%)	200 (20.2)	150 (9.9)	< 0.001	79 (16.4)	55 (11.4)	0.032
Acute urinary retention (CD1)	45 (4.5)	39 (2.6)	0.010	18 (3.7)	11 (2.3)	0.258
Prolonged irrigation for haematuria (CD2)	24 (2.4)	55 (3.6)	0.119	13 (2.7)	28 (5.8)	0.025
Blood transfusion (CD2)	5 (0.5)	5 (0.3)	0.719	2 (0.4)	2 (0.4)	> 0.99
Bleeding requiring surgical control (CD3)	8 (0.8)	14 (0.9)	0.937	2 (0.4)	8 (1.7)	0.112
Urinary tract infection (UTI) (CD2)	78 (7.9)	19 (1.2)	< 0.001	29 (6.0)	3 (0.6)	< 0.001
Sepsis needing ICU (CD4)	3 (0.3)	3 (0.2)	0.911	2 (0.4)	0	0.479
Secondary morcellation (CD3)	17 (1.7)	1 (0.1)	< 0.001	5 (1.0)	0	0.073
Ureteric orifice injury needing stenting(UO), (CD3)	2 (0.2)	2 (0.1)	> 0.99	0	1 (0.2)	> 0.99
Cardiovascular complications (CD4)	2 (0.2)	6 (0.4)	0.635	0	0	–
Prolonged bleeding with need for additional haemostasis (CD3)	7 (0.7)	4 (0.3)	0.182	4 (0.8)	2 (0.4)	0.682
Energy device/morcellator malfunction	2 (0.2)	2 (0.1)	> 0.99	0	0	–
Minor bladder injury from morcellation (CD2)	4 (0.4)	2 (0.1)	0.343	4 (0.8)	2 (0.4)	0.682
Redo surgery within 30 days (CD4)	1 (0.1)	0 (0.0)	0.829	0	0	–
Postoperative incontinence, *n* (%)	156 (15.7)	168 (11.0)	0.001	86 (17.8)	77 (16.0)	0.492
Type of incontinence, *n* (%)						
Urge	23 (2.3)	31 (2.0)	0.736	11 (2.3)	17 (3.5)	0.338
Stress	97 (9.8)	95 (6.2)	0.001	56 (11.6)	39 (8.1)	0.084
Mixed	29 (2.9)	34 (2.2)	0.341	18 (3.7)	18 (3.7)	> 0.99
Duration of incontinence for those affected, *n* (%)						
< 1 month	72 (48.6)	88 (55.7)	0.407	40 (47.6)	43 (58.1)	0.378
1–3 months	49 (33.1)	48 (30.4)		32 (38.1)	21 (28.4)	
> 3 months	27 (18.2)	22 (13.9)		12 (14.3)	10 (13.5)	
Kegel exercise needed, *n* (%)	108 (77.1)	145 (86.3)	0.052	64 (80.0)	68 (88.3)	0.228
30-day readmission, *n* (%)	20 (2.0)	33 (3.1)	0.159	10 (2.1)	17 (3.5)	0.242
Delayed complications, *n* (%)	22 (2.2)	21 (2.0)	0.814	11 (2.3)	12 (2.5)	> 0.99
Urethral stricture requiring dilation only	7 (0.7)	10 (0.9)	0.734	4 (0.8)	6 (1.2)	0.751
Urethral stricture requiring urethrectomy	6 (0.6)	8 (0.8)	0.893	3 (0.6)	4 (0.8)	> 0.99
Bladder neck stenosis requiring BNI	8 (0.8)	2 (0.2)	0.089	4 (0.8)	1 (0.2)	0.370
Stress incontinence requiring sling	1 (0.1)	1 (0.1)	> 0.99	0	1 (0.2)	> 0.99

*IDC *Indwelling catheter, *BNI *bladder neck incision, *CD *Clavien-Dindo

Only 1.96% of the PSM cohort had UI that persisted over 3 months. Univariate analysis revealed that age, prostate volume, pre-operative Qmax, pre-operative post-void residual urine (PVRU) and total operative time were predictors of UI (Table 4). With the exception of prostate volume, all these parameters were found to be significant predictors on multivariate analysis (Table 5).

**Table 4:** Univariate analysis of risk factors for incontinence

	PSM cohort		
	OR	95% CI	*p*
En-bloc (vs non-en-bloc)	0.875	0.624–1.230	0.440
Age	1.031	1.007–1.055	0.011
Prostate volume (vs 80–100 ml)			
101–200 ml	0.464	0.235–0.968	0.032
> 200 ml	0.384	0.156–0.947	0.036
Preoperative indwelling catheter			
Preoperative IPSS	0.967	0.928–1.008	0.116
Preoperative QOL	1.218	1.001–1.491	0.052
Preoperative Qmax	0.88	0.817–0.946	0.001
Preoperative PVRU	1.004	1.002–1.005	< 0.001
Energy source (vs low-power holmium)			
High-power holmium laser	0.574	0.268–1.341	0.172
Holmium laser with MOSES	1.264	0.457–3.57	0.652
Thulium fiber	0.692	0.317–1.642	0.376
Thulium-YAG	0.625	0.125–2.444	0.523
Bipolar enucleation	0.726	0.329–1.733	0.445
Monopolar enucleation	0.37	0.019–2.393	0.375
Early apical release	0.869	0.609–1.251	0.443
Total operation time	1.009	1.006–1.013	< 0.001

*IPSS *International prostate symptom score, *QOL *quality of life, *Qmax *maximum flow rate, *PVRU *post void residual volume

**Table 5:** Multivariate adjusted odds ratio of incontinence in the PSM cohort

	PSM cohort		
	OR	95% CI	*p*
En-bloc (vs non-en-bloc)	0.899	0.653–1.235	0.511
Age	1.024	1.004–1.045	0.018
Prostate volume (vs 80–100 ml)			
101–200 ml	0.722	0.437–1.236	0.217
> 200 ml	0.686	0.342–1.366	0.283
Preoperative Qmax	0.94	0.890–0.990	0.022
Preoperative PVRU	1.003	1.002–1.005	< 0.001
Total operation time	1.006	1.003–1.010	< 0.001

## Discussion

In the current era, AEEP has become a well-established intervention for large (> 80 cc) prostate burdens. In this setting, it offers a more favourable peri-operative safety profile compared to OSP [1]. However, continued research is needed to develop consensus regarding the best technique and energy source along with other specifications for follow-up as was highlighted in the Refine Endoscopic Anatomical Enucleation of the Prostate (REAP) registry [10]. Most experts now agree that the urethral sphincter should be detached at the start of enucleation, referred to as the EAR manoeuvre as well identifying the correct surgical plane [11]. AEEP is recognised as a more technically challenging procedure compared to alternatives such as transurethral resection of the prostate (TURP). To this end, European guidelines outline that it is surgeon experience that has the largest impact on complications and mentorship programmes are recommended accordingly [1, 12, 13].

A recent randomised trial by Shoma et al. compared holmium laser vs. thulium laser vs. bipolar enucleation in 155 patients with prostate size > 80 cc [14]. Their results revealed no differences in IPSS, QoL, or PVR at 12 months follow-up and their findings support the conclusion that AEEP is influenced more by technique than energy source. A finding that was supported in a real world study comparing TFL with High-power Holmium was that urologists should focus on performing good anatomic removal of prostate tissue, with the choice of laser being not as important for outcomes and complications can occur even in the hands of experienced surgeons [15]. This is also pertinent to EEP in large prostates. Interestingly, the authors in a recent systematic review recorded better postoperative functional outcomes in prostates with a volume of ≥ 175, > 200 and > 300 ml, respectively, with a retreatment rate of only 0–1.3% [16]. However, the authors could not make deductions from the pooled data on which technique is better for large and very large prostates. Rucker et al. reported that all Holmium enucleation techniques show similar postoperative outcomes but en-bloc and two-lobe enucleation are significantly faster with respect to enucleation, overall operation time, and speed compared to the three-lobe technique, but this did not have large prostates only [17]. In our study, enucleation time was clearly in favour of the en-bloc technique for large and very large prostates yet morcellation times were slower as was also shown in a study by Enikeev et al. especially in prostates > 150 cc [18]. It is, therefore, not the technique but the visibility in large, often vascular prostates, which may be contributory to slower morcellation [19]. We also acknowledge that the type of morcellator and surgical setup influences these outcomes too [20]. Further evaluation is needed to investigate the differences in enucleation and total operative time between the techniques.

Recently, in a study by Tricard et al. which evaluated outcomes associated with endoscopic enucleation in prostates > 150 cc, concluded that OSP is ‘dead’ [2, 21]. The primary endpoint was the success of the procedure, defined by a complete endoscopic enucleation of the prostate, absence of blood transfusion or reoperation for bleeding, post-operative improvement of quality of life (assessed by a ≥ 2 points increase in the 8th question of the IPSS test) and post-operative continence (no pad use) at 3 months follow-up.

In our series, none of the patients in PSM cohort required re-do surgery. In the non-PSM cohort, seven patients needed blood transfusion and two in the en-bloc group. On PSM, this was balanced out in both. The en-bloc group had no cases of sepsis and a lower incidence of post-operative acute urinary retention.

While the incidence of persistent UI post AEEP is relatively low (1–5%), rates of transient UI, which are predominantly of the stress variant, are higher compared to post-TURP [22]. This is especially the case for beginners. In our study, non-en bloc vs en-bloc status had no significant effect on the rate of post-operative UI. A recent consensus project, which addressed technique standardisation, recommended preservation of apical mucosa and judicious use of energy at this site as well as only gentle disruption of the lateral lobes at their apices [11]. Novel additions to standard surgical steps have been reported by Huang et al. The authors recorded outcomes associated with injecting of intradetrusor onabotulinumtoxinA (Botox^®^) in patients undergoing Holmium enucleation who also experienced severe storage symptoms. The authors found it resulted in significant reduction in incontinence scores at 3 months [23]. Partial adenectomy e.g. median lobe enucleation only has also been put forward as a means to reduce rates of post-operative UI [24]. Post-operative pelvic floor muscle training in the form of Kegel exercises were a routine part of this study but their role as part of AEEP rehabilitation remains a subject of debate. We noted that in our study as well, an equal number of patients were advocated Kegel’s exercise. A recent randomised study by Anan et al. suggest that such exercises significantly reduced UI at 3 months (3% vs. 26%, *p* = 0.01) follow-up, but made no difference at long-term follow-up 0% vs. 3%; *p* = 1.00 [25].

## Strengths and limitations

There are certain drawbacks in this study to acknowledge. Firstly, the retrospective nature introduces bias. One example of this is that it is not possible to fully determine why a particular technique was adopted and whether it was decided pre-operatively e.g. due to surgeon preference or intraoperative e.g. based on emergency clinical factors. Our study did not include certain parameters such as frailty, obesity or catheter dependency, which other studies have found to be predictors of post-operative incontinence [26]. This study was strengthened by including data from nine centres and a large sample, which allowed for a well-matched propensity cohort to be established. However, long-term longitudinal follow-up was lacking as is not uncommon when data are collected from tertiary centres, where patients may have their care taken over by local centres [24]. Protocols regarding for example antibiotic prophylaxis vary across sites and is an inherent limitation in such multi-centre studies with pooled data. We acknowledge that underreporting may have been possible that could influence the outcomes of this study. No information is available on how the incontinence measures were documented and what questionnaires were used. However, there is merit that these are well-established high-volume centres with experienced urologist sharing their data on large and very large prostates. Therefore, we rely on their experience to provide useful data that attempts to throw light on peri- and post-operative outcomes of the different techniques deployed. Another factor that may have influenced outcomes is the surgeon’s own adaptation of the technique. We are also limited by the lack of post-operative weight of the resected tissue and follow-up PSA to comment on efficiency and completeness of surgery, as this can be a surrogate to functional outcomes. Another limitation is non availability of information on anticoagulant management. We think that our limitations can prompt future studies to ensure that all these missing parameters to be considered when designing future trials including having the technique approach assigned before surgery. We can make some inferences but acknowledge that for all conclusive deductions prospective and comparative studies are needed to further evaluate the formal role of en-bloc technique on AEEP outcomes. It is an advantage that this large real world cohort of cases was done across different health systems, was PSM matched and the volume of cases throws useful insights validating most findings of single centre studies. The findings regarding the of early apical release and lack of differences in continence outcomes are also novel and timely given the increased attention in this area.

## Conclusion

In experienced hands, AEEP in large prostates by the en-bloc technique yields a lower rate of complication and has a slightly shorter operative time compared to the non en-bloc approach. This might benefit surgeons with high case yield per operative list. However, beyond 80 cc, neither prostate size nor technique had any influence in rates of post-operative incontinence for large and very large prostates. Whilst we are unable to say which energy is the best for any surgical approach, age, pre-operative Qmax, pre-operative PVRU and total operative time were all predictors of incontinence. Reassuringly, this incontinence is temporary and seldom lasts beyond 3 months.

## Data Availability

The data sets generated during and/or analyzed during the current study are available from the corresponding author on reasonable request.
